# The effectiveness and cost-effectiveness of screening for active tuberculosis among migrants in the EU/EEA: a systematic review

**DOI:** 10.2807/1560-7917.ES.2018.23.14.17-00542

**Published:** 2018-04-05

**Authors:** Christina Greenaway, Manish Pareek, Claire-Nour Abou Chakra, Moneeza Walji, Iuliia Makarenko, Balqis Alabdulkarim, Catherine Hogan, Ted McConnell, Brittany Scarfo, Robin Christensen, Anh Tran, Nick Rowbotham, Teymur Noori, Marieke J van der Werf, Kevin Pottie, Alberto Matteelli, Dominik Zenner, Rachael L Morton

**Affiliations:** 1 Division of Infectious Diseases, Jewish General Hospital, McGill University, Montreal, Canada; 2 Centre for Clinical Epidemiology of the Lady Davis Institute for Medical Research, Jewish General Hospital, Montreal, Canada; 3 Department of Infection, Immunity and Inflammation, University of Leicester, Leicester, United Kingdom; 4 Department of Microbiology and Infectious Diseases, Université de Sherbrooke, Québec, Canada; 5 Musculoskeletal Statistics Unit, The Parker Institute, Bispebjerg and Frederiksberg Hospital, Copenhagen, Denmark; 6 National Health and Medical Research Council (NHMRC) Clinical Trials Centre, University of Sydney, Sydney, Australia; 7 European Centre for Disease Prevention and Control, Stockholm, Sweden; 8 C.T. Lamont Primary Health Care Research Centre, Bruyère Research Institute, Ottawa, Ontario, Canada; 9 Clinic of Infectious and Tropical Diseases, University of Brescia and Brescia Spedali Civili General Hospital, World Health Organization Collaborating Centre for TB/HIV and TB Elimination, Brescia, Italy; 10 Respiratory Diseases Department, Centre for Infectious Disease Surveillance and Control (CIDSC), Public Health England, London, United Kingdom; 11 Department of Infection and Population Health, University College London, London, United Kingdom

**Keywords:** active tuberculosis, migrants, screening, EU/EEA

## Abstract

: The foreign-born population make up an increasing and large proportion of tuberculosis (TB) cases in European Union/European Economic Area (EU/EEA) low-incidence countries and challenge TB elimination efforts. **Methods** : We conducted a systematic review to determine effectiveness (yield and performance of chest radiography (CXR) to detect active TB, treatment outcomes and acceptance of screening) and a second systematic review on cost-effectiveness of screening for active TB among migrants living in the EU/EEA. **Results** : We identified six systematic reviews, one report and three individual studies that addressed our aims. CXR was highly sensitive (98%) but only moderately specific (75%). The yield of detecting active TB with CXR screening among migrants was 350 per 100,000 population overall but ranged widely by host country (110–2,340), migrant type (170–1,192), TB incidence in source country (19–336) and screening setting (220–1,720). The CXR yield was lower (19.6 vs 336/100,000) and the numbers needed to screen were higher (5,076 vs 298) among migrants from source countries with lower TB incidence (≤ 50 compared with ≥ 350/100,000). Cost-effectiveness was highest among migrants originating from high (> 120/100,000) TB incidence countries. The foreign-born had similar or better TB treatment outcomes than those born in the EU/EEA. Acceptance of CXR screening was high (85%) among migrants. **Discussion**: Screening programmes for active TB are most efficient when targeting migrants from higher TB incidence countries. The limited number of studies identified and the heterogeneous evidence highlight the need for further data to inform screening programmes for migrants in the EU/EEA.

## Introduction

Tuberculosis (TB) is a public health priority in the European Union (EU) and European Economic Area (EEA), and countries have committed themselves to the World Health Organization (WHO) *End TB Strategy* with an ambitious goal to end TB [1-4]. The foreign-born population make up an increasing and considerable number and proportion of all TB cases in countries with low TB incidence (< 10 cases/100,000 population) and challenge TB elimination efforts in the EU/EEA [3,5]. More than one quarter of reported TB cases in 2015 in the EU/EEA occurred in the foreign-born population [5]. This proportion has been increasing steadily; in 2007, 13.6% of TB cases occurred in migrant populations whereas in 2013, they accounted for 21.8% [6]. In many low TB incidence countries in the EU/EEA, more than half of all TB cases occur among foreign-born individuals [5]. Between 2007 and 2012, the EU/EEA received on average 1.5 million migrants from outside of the EU/EEA, and larger numbers in 2015 and 2016 [7,8]. As a result, the foreign-born population now makes up 11.4% of the population in the EU/EEA and exceeds 15% in many low TB incidence countries [7,8]. A considerable proportion of these migrants were born in countries with a high TB burden [9,10].

Given the disproportionate TB case notifications in migrant populations and the faster decline of TB rates in host populations, enhanced TB control strategies among migrants will be necessary to achieve TB elimination in the EU/EEA (defined as achieving a rate of less than one case of TB per 1,000,000 population) [1-4,11,12]. Countries have generally focused on two targeted control strategies among migrants: (i) identification of active TB with chest radiography (CXR) before or soon after arrival in the host country to detect prevalent TB cases and limit onward transmission and (ii) more recently, identifying and treating latent TB in migrants from high TB burden countries to prevent TB reactivation [13]. Many EU/EEA countries with low TB incidence screen migrants for active TB on or soon after arrival. The migrant groups targeted for screening and the location of screening are different for each country because screening guidelines for active TB in migrants are lacking at the EU/EEA level [13-15]. We conducted a systematic review on the effectiveness and a second systematic review on the cost-effectiveness of screening for active TB among migrants in the EU/EEA region with the aim of informing migrant screening guidelines.

## Methods

### Overall approach and key questions

**Figure 1:**
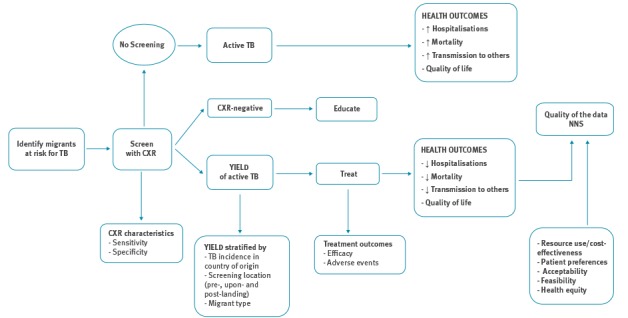
Analytic framework of the evidence chain for active tuberculosis screening in migrants

CXR: chest radiography; NNS: number needed to screen; TB: tuberculosis.

This review supports a project of the European Centre for Disease Prevention and Control (ECDC) to develop guidance on screening for six infectious diseases (chronic hepatitis C, hepatitis B, HIV, TB (active and latent) and intestinal parasites) in newly arrived migrants to the EU/EEA. The project followed the new Grading of Recommendations Assessment, Development and Evaluation (GRADE)-ADOLOPMENT approach to conduct systematic reviews on screening migrant populations for these six infectious diseases [16]. The review protocol and the methods of GRADE-ADOLOPMENT guideline development have been published [16,17]. All reviews followed a Cochrane methodological approach and the Preferred Reporting Items for Systematic Reviews and Meta-Analyses (PRISMA) methods for reporting systematic reviews [18]. For each review, we developed two research questions (using a population, intervention, comparison and outcome (PICO) framework), an analytic framework to illustrate the screening evidence pathway, and identified and prioritised clinically important outcomes, following the evidence-based review methods described by the United States (US) Preventative Task Force [19,20]. We sought to answer two research questions: (i) what is the effectiveness of screening migrants arriving and living in the EU/EEA for active TB and (ii) what is the resource use, cost and cost-effectiveness of screening migrants for active TB? We developed an analytic framework that identified the evidence chain to address the effectiveness and cost-effectiveness of active TB screening among migrants (Figure 1) [17]. We developed the following key questions along this evidence chain: (i) what is the yield of active TB screening with CXR in migrants, (ii) what are the test performance characteristics of CXR to detect active TB, (iii) how effective is active TB therapy and what are the associated harms, (iv) what is the uptake of active TB screening by migrants, and (v) how cost-effective is screening for active TB in migrants [17]?

### Search strategy and selection criteria

Following the GRADE-ADOLOPMENT process, we identified an evidence review that assessed the effectiveness of latent TB infection (LTBI) screening among migrants, published in 2011 by the Canadian Collaboration on Immigrant and Refugee Health (CCIRH), and used this as a starting point for our literature search (anchoring review) [16,21]. The CCIRH review included systematic reviews on the effectiveness of LTBI screening in migrants up to 2008 but did not review cost-effectiveness. We therefore conducted two separate searches to address our research questions. The first search updated the CCIRH evidence review and identified systematic reviews and guidelines on the effectiveness and cost-effectiveness of TB screening programmes in migrant populations from 2005 to 2016. The second search identified individual studies on the resource use, costs and cost-effectiveness of TB screening programmes for migrants over a longer period, 2000 to 2016, given these topics were not covered in the CCIRH evidence review. For the first search, MEDLINE via Ovid, Embase, the Cumulative Index to Nursing and Allied Health Literature (CINAHL), Epistemonikos and Cochrane CENTRAL between 1 January 2005 and 12 May 2016 were searched. We used a combination of key terms including: ‘tuberculosis’, ‘screening’, ‘chest-radiograph’, ‘tuberculin skin test’, ‘interferon-gamma release assays’, ‘costs’, ‘cost-effectiveness’ AND ‘guidelines’ and ‘reviews’. The search terms and strategy in Ovid MEDLINE are included in Supplement 1. We also searched grey literature websites for published guidelines and reports from the US Centres for Disease Control and Prevention (CDC), ECDC, WHO and the International Union Against Tuberculosis and Lung Disease (IUATLD). We did not apply language restrictions to the search. Additional guidelines and studies were identified by our co-authors and through searching bibliographies of included studies. In the second search, using the search terms ‘tuberculosis’, ‘screening’, ‘costs’ and ‘cost-effectiveness’, we searched MEDLINE, Embase, the National Health Service Economic Evaluation Database (NHS EED), the Database of Abstracts of Reviews of Effects (DARE), the Tufts Medical Center Cost-Effectiveness Analysis Registry and Google Scholar for entries between 1 January 2000 and 31 May 2016.

### Study selection and quality assessment

We identified and included systematic reviews and evidence-based guidelines that directly addressed each key question along the active TB screening evidence chain and prioritised documents focusing on newly arrived (< 5 years in the host country) migrants. Migrant populations included were non-forced economic migrants, and refugees, asylum seekers and illegal migrants who may have been forced to flee conflict, natural disaster, or economic peril [17]. We only included studies published in full and in English or French. If more than one version of a systematic review was identified, the most recent was considered. Studies were excluded if they were not relevant to the key questions, if they were not a systematic review or guideline, if the study methodology was unclear, and if they focussed only on non-generalisable subgroups (such as healthcare workers or HIV-positive people) or addressed only latent TB screening. Two authors screened the titles and abstracts, assessed selected full-text articles for eligibility and extracted data from included articles. Disagreements were resolved by consensus or by a third author. The methodological quality of systematic reviews was assessed using the AMSTAR tool (A Measurement Tool to Assess Systematic Reviews) and the quality of individual studies was assessed with the Newcastle-Ottawa scale [22,23]. The GRADE criteria were applied to assess the quality and certainty of the evidence for the individual studies included in the systematic reviews [24].

### Data extraction and synthesis

The following information was extracted from each study: study design, objectives, analyses, quality assessment of the individual studies included in the systematic review, population examined, number of included studies, total number of participants included, intervention, outcome and results. We created GRADE evidence profiles and summary of findings tables for each outcome where appropriate. Numbers needed to screen (NNS) were estimated by calculating 1/mean prevalence of active TB found through CXR screening stratified by TB incidence in the country of origin as reported in the study by Aldridge et al. [25].

For each of the cost-effectiveness studies, we extracted the following data: economic methods used (e.g. micro-costing study, within-trial cost-utility analysis, Markov model), description of the case base population, the intervention and comparator, the absolute size and relative difference in resource use and cost-effectiveness (e.g. incremental net benefit (INB) or incremental cost-effectiveness ratio (ICER)) [26]. The certainty of economic evidence in each study was assessed using the relevant items from the 1997 Drummond checklist [27]. All currencies were converted to 2015 Euros using the Cochrane web-based currency conversion tool: https://eppi.ioe.ac.uk/costconversion/default.aspx.

## Results

**Figure 2:**
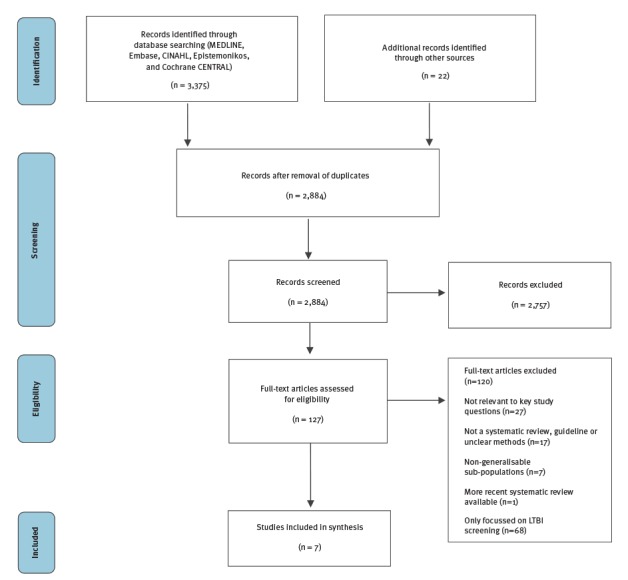
PRISMA flow diagram, literature search for the effectiveness and cost-effectiveness of active tuberculosis screening, 1 January 2005–12 May 2016

CINAHL: Cumulative Index to Nursing and Allied Health Literature; LTBI: latent tuberculosis infection.

**Figure 3:**
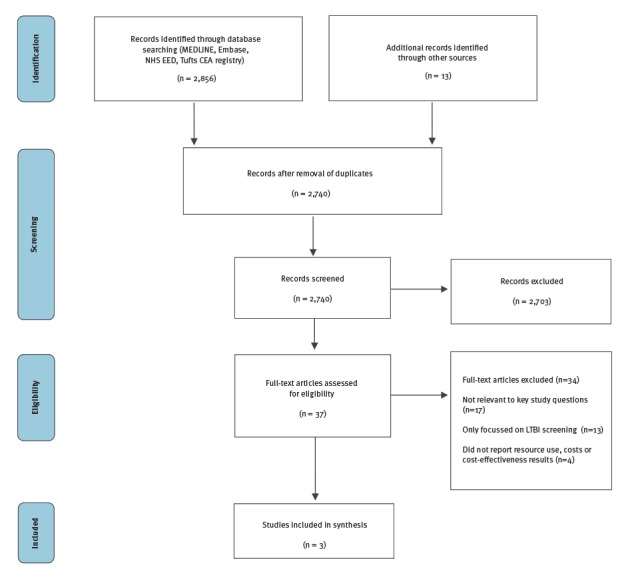
PRISMA flow diagram, literature search for the resource use, costs and cost-effectiveness of active tuberculosis screening, 1 January 2000–31 May 2016

In the first search, we retrieved 3,375 studies through database searching and 22 additional studies identified through other sources on the effectiveness of TB screening in migrant populations (Figure 2). After removal of duplicates, 2,884 studies were screened by title and abstract. A total of 127 studies underwent full text assessment. We did not identify any single study on the effectiveness of active TB screening in migrants. We therefore included seven studies that addressed the active TB screening evidence chain: the yield of detecting active TB among migrants in CXR screening programmes (n = 3) [25,28,29], the performance characteristics of CXR to detect active TB (n = 2) [30,31], the effectiveness of TB therapy in those born in the EU/EEA and the foreign-born population (n = 1) [6], and the uptake of active TB screening by migrants (n = 1) [32]. In the second search, 2,856 articles were retrieved through database searching and an additional 13 articles identified through other resources (Figure 3). After removal of duplicates, 2,740 studies were screened by title and abstract. A total of 37 studies underwent full text assessment and three individual studies were included for analysis [33-35].

LTBI: latent tuberculosis infection; NHS EED: National Health Service Economic Evaluation Database; Tufts CEA: Tufts Medical Center Cost-Effectiveness Analysis Registry.

### Effectiveness of active tuberculosis screening

#### Yield of chest radiography to detect active tuberculosis 

Three systematic reviews assessed the yield of detecting active TB among migrant populations in CXR screening programmes performed before and after arrival in the EU/EEA and low TB incidence countries outside the EU/EEA [25,28,29]. The yield of active TB was heterogeneous across studies, varied by migrant type and the setting in which the screening was done and was consistently higher with higher TB incidence in the country of origin (Table 1). 

**Table 1:** Characteristics of included studies for effectiveness of active tuberculosis screening

Study	Certainty of evidence	Design	Population	Intervention/outcomes	Results
Klinkenberg et al. 2009 [29]	Quality of systematic review (AMSTAR): 3/11. Quality of data of included individual studies (GRADE): low.	Systematic review 1998–2008. Observational studies: EU/EEA (n = 36), non-EU (n = 14). EU countries included: Belgium, Denmark, France, Germany, Greece, Ireland, Italy, the Netherlands, Norway, Spain, Switzerland, UK.	New entrants to the EU/EEA: migrant, asylum seeker, foreign-born citizen, illegal foreigner/migrant. Non-EU were performed in the US, Canada, Australia and Japan. Type of screening: mandatory (n = 24,156), voluntary: (n = 2,855). Type of migrant: asylum seekers: (n = 17,824), other migrants: (n = 5,925), migrants/asylum seekers (n = 218,565).	Intervention: screening by CXR (at port of arrival, reception/holding/transit centre, community post-arrival, occasional screening, follow-up screening). Outcomes: yield of active TB/100,000, 95% CI, median and IQR.	Median active TB yield/100,000, (IQR): EU countries: 350 (110–710), non-EU countries: 510 (170–1,230). Screening type: mandatory (EU): 280 (100–420); voluntary (EU): 400 (160–980). Migrant type (EU): asylum seeker: 350 (250–410), other migrant: 170 (100–630), migrant/asylum seeker: 300 (9–500). Screening setting (EU): port of arrival: 360 (100–520), port of arrival and community post arrival: 650 (0–0), reception/holding centre: 290 (100–380), community post arrival: 220 (100–380), follow-up: 120 (90–170), occasional: 1,720 (730–2,740), port of arrival and occasional: 720 (710–1,000).
Arshad et al. 2010 [28]	Quality of systematic review (AMSTAR): 7/11. Quality of data of included individual studies (GRADE): low–very low.	Systematic review up to July 2008. Observational studies (n = 22). EU countries included: Belgium Denmark, Ireland, the Netherlands, Norway, Spain, Switzerland, UK.	Migrants assessed through active case finding or active screening programme irrespective of symptoms. n = 5,446 pulmonary TB, n = 2,620,739 screened migrants. Total types of migrants screened: asylum seekers (n = 135,265), regular immigrants (n = 2,466,492), refugees (n = 18,982).	Intervention: CXR and/or sputum smear and/or microbiological culture; routine screening programmes/on purpose screening. Outcome: number of cases detected per 100,000 individuals screened (95% CI). RR: pooled prevalence for pulmonary tuberculosis among screened migrants compared with general population in host country (95% CI).	Active TB yield/100,000 (95% CI): 349 (290–408); RR (95% CI): 48.2 (23.3–99.6). Immigrant class: refugees: 1,192 (668–1,717); RR 130.6 (58.8–290.2), migrants: 284 (204–364); RR 29.4 (9.7- 88.9), asylum seekers: 270 (198–342); RR 30.1 (19.3–47.1). European countries/immigrant class: refugees: 577 (206–949), migrants: 225 (129–322), asylum seekers: 267 (194–341). Region of origin: Europe: 236 (131–340), Africa: 655 (319–990), Asia: 1,117 (625–1,608).
Aldridge et al. 2014 [25]	Quality of systematic review (AMSTAR): 8/11. Quality of data of included individual studies (GRADE): very low.	Systematic review 1980–2014. n = 15 studies.	Migrants, asylum seekers, foreign-born citizens, undocumented foreigners or migrants. 3,739,266 migrants screened between 1982 and 2010: min 873– max 3,092,729 culture-confirmed. Types of migrants screened: migrants (n = 592,673); refugees (n = 52,991), mixed (n = 3,092,729), adoptees: n = 873).	Interventions: CXR, culture, smear for acid-fact bacilli, drug-resistant disease, LTBI (any method). Outcome: yield of culture-confirmed active TB per 100,000 by TB prevalence in country of origin.	TB incidence/100,000 person-years at 7 years post migration: Africa: 190, Asia: 80, Somalia: 520, Pakistan: 160, Vietnam: 210, Former Yugoslavia: 40/100,000.
Van’t Hoog et al. 2013 [30]	Quality of systematic review (AMSTAR): 6/11. Quality of data of included individual studies (GRADE): very low.	Systematic review 1992–2012. n = 17 studies (24 publications), 11 community prevalence surveys.	Adults (> 15 years) or general population undergoing first screening (HIV-negative and unknown HIV status). Median: 8,044 participants, IQR: 98–20,566.	Intervention: symptoms, CXR, combinations. Outcomes: sensitivity and specificity (95% CI) to detect active TB.	CXR screening had greater accuracy compared with symptoms screening. CXR with any abnormality: sensitivity (95% CI): 97.8% (95.1–100.0), specificity (95% CI): 75.4% (72.0–78.8). CXR with abnormality suggestive of TB: sensitivity: 86.8% (79.2–94.5), specificity: 89.4% (86.7–92.0). Any symptom screening: High HIV/SSA: sensitivity: 84.2% (75.6–92.7), specificity: 74.0% (53.1–94.9). Low HIV/Asia: sensitivity: 69.8% (57.9–81.8), specificity: 60.6% (34.7–86.0). Low and high HIV combined: sensitivity: 77.0% (68.0–86.0), sensitivity: 67.7% (50.2–85.1).
Pinto et al. 2013 [31]	Quality of systematic review (AMSTAR): 8/11. Quality of data of included individual studies not mentioned but all studies had verification bias (assessed by QUADAS): 54% not representative, 46% did not mention blinding.	Systematic review up to 2012. n = 12 studies with combined clinical and radiographic features, 1 with clinical prediction rules.	Adult patients (≥ 15 years) with possible PTB (excluding pneumoconiosis, malignancies, immune-mediated inflammatory disease or haemodialysis). 5,767 participants.	Intervention: CXR scoring system. Outcomes: sensitivity and specificity (95% CI) with no pooling (median, range presented), diagnostic OR: odds of patient with PTB and specific clinical or radiographic feature(s)/odds without PTB and having the same feature(s).	Significantly associated with pulmonary TB: upper lobe infiltrates: OR (95% CI): 3.57 (2.38–5.37), cavities diagnostic: OR range: 1.97–25.66. Scoring systems characteristics: sensitivity: median 96%, IQR: 93–98%, sensitivity: median 46%, IQR: 35–50%.
Ködmön et al. 2016 [6]	High quality individual study (assessed by New Castle-Ottawa): 8/8.	Public health surveillance of reported active TB cases from EU and EEA countries 2007–2013. 29 countries.	Notified TB cases. 527,467 TB cases reported, 491,652 with reported country of origin, 91,925 cases from outside EU/EEA.	Intervention: N/A. Outcomes: successful treatment: cured case or treatment completed after 12 months, death during treatment.	Number of reported TB treatment outcome: EU/EEA: 86%, non-EU/EEA: 82%. Treatment success (24 countries): EU/EEA: 74.6%, non-EU/EEA: 77.4%. Treatment failure: EU/EEA: 2.3%, non-EU/EEA: 0.2%. Lost to follow-up: EU/EEA: 6.6%, non-EU/EEA: 5.4%. Death during treatment: EU/EEA: 8.2%, non-EU/EEA: 3.2%.
Mitchell et al. 2013 [32]	Quality of systematic review (AMSTAR): 3/11. Quality of studies judged to have significant degree of heterogeneity and reporting and publication bias. The tool used to measure bias was not mentioned.	Qualitative and quantitative systematic review and meta-synthesis. n = 218 studies.	(i) Risk groups found in health services (adolescents, drug-dependent, HIV-positive etc.). (ii) Congregate/occupational/environmental (elderly, HCWs, prisoners etc.). (iii) Behavioural/marginalised risk groups (homeless, migrants, sex workers etc). 33 possible risk groups.	Intervention: N/A. Outcome: proportion of eligible persons who consented to undergo TB screening, per risk-group (equivalent of recruitment rate).	TB screening acceptability: overall: > 80%, migrants: 85% (range: 55–96%). Simple TB screening (at point-of-care) more acceptable than referral on multiple visits. Inclusion of HIV testing may be a deterrent in some risk groups. TB screening and treatment are low priority for groups facing housing insecurity, addiction, threat of violence, deportation. Screening in hard-to-reach populations is more acceptable if benefits are immediate and tangible. Acceptability of TB screening is dependent on quality of human interaction as well as perceived negative consequences.

AMSTAR: A MeaSurement Tool to Assess systematic Reviews [22]; CI: confidence interval; CXR: chest radiography; EEA: European Economic Area; EU: European Union; GRADE: The Grading of Recommendations Assessment, Development and Evaluation; HCW: healthcare workers; HIV: human immunodeficiency virus; IQR: interquartile range; LTBI: latent tuberculosis infection; N/A: not applicable; OR: odds ratio; PTB: pulmonary TB; QUADAS: Quality Assessment of Diagnostic Accuracy Studies; RR: risk ratio; SSA: sub-Saharan Africa; TB: tuberculosis; UK: United Kingdom; US: United States.

Klinkenberg et al. found that the overall yield of active TB screening programmes in migrants upon and after arrival in 26 studies done in EU/EEA countries was 350 per 100,000 population [29]. The yield differed by migrant type (asylum seekers: median: 350/100,000; interquartile range (IQR): 250–410, and other migrants: median: 170; IQR: 100–630) and by setting where the screening was conducted (port of arrival: median: 360; IQR: 100–5,200, reception/holding centres: median: 290; IQR: 100–380, community post arrival: median: 220; IQR: 100–380, and occasional screening: median: 1,720; IQR: 730–2,740). The yield varied widely also between host countries, from as low as 110 per 100,000 in the Netherlands to as high as 2,340 per 100,000 in Italy, probably reflecting differences in migrant type, country of origin and circumstances of travel in the migrants screened [36]. Arshad et al. assessed the yield of active TB screening among migrants originating from intermediate or high TB incidence countries upon and after entry to low TB incidence countries and found a similar overall yield of active TB case detection of 349 per 100,00 population [28]. The yield also varied by migrant type (refugees: 1,192; 95% confidence interval (CI): 668–1,717, regular migrants: 284; 95% CI: 204–364 and asylum seekers: 270; 95% CI: 198–342) and TB incidence in the country of origin (Europe: 236; 95% CI: 131–340, Africa: 655; 95% CI: 319–990 and Asia: 1,117; 95% CI: 625–1,608) [28]. Finally, Aldridge et al. assessed the yield of CXR screening for active TB among migrants in the pre-arrival TB screening programmes. No overall estimates were presented but the yield increased steadily with the TB incidence in migrant source countries. The yield was 19.6 per 100,000 in migrants originating from countries with a TB incidence lower than 50 per 100,000 and 336 per 100,000 in migrants originating from countries with a TB incidence greater than 350 per 100,000 [25]. The quality of the data in studies included in these three systematic reviews was very low to low (GRADE).

#### Accuracy of chest radiography to detect active tuberculosis

We identified two systematic reviews that assessed the performance of CXR to detect active TB [30,31]. Van’t Hoog et al. showed that CXR (presence of any abnormality) was highly sensitive (98%) and moderately specific (75%) to detect active TB [30]. Screening for active TB with symptoms alone had lower sensitivity (78%) and specificity (68%) [30]. Pinto et al. also found that CXR to detect active TB was highly sensitive 95% (range: 81–100%) but less specific 42% (range: 22–72%) [31]. Focussing on the presence of upper lobe infiltrates and cavities increased the predictive value for diagnosing active TB. The certainty of the evidence of these two studies was judged to be very low (Table 1).

#### Numbers needed to screen

Using inputs of the yield of CXR reported by Aldridge in the pre-arrival programmes we estimated the NNS to detect one case of active TB in migrants stratified by TB incidence in source countries (Table 2) [25]. We found that the NNS decreased dramatically with increasing TB incidence in source countries and ranged from 5,076 in countries with a TB incidence between 50 and 149 per 100,000 to 298 in countries with a TB incidence greater than 350 per 100,000.

**Table 2:** Numbers needed to screen to detect one case of active tuberculosis

TB prevalence in country of origin/100,000	Yield of culture-confirmed active TB/100,000^a^	95% CI	NNS ^b^	95% CI
50–149	19.7	10.3–31.6	5,076	3,175–9,709
150–249	166.2	140–194	602	514–714
250–349	133.5	111–158	749	631–903
> 350	335.9	283–393	298	254–353

CI: confidence interval; CXR: chest radiography; NNS: numbers needed to screen; TB: tuberculosis.

^a^ The yield of active TB detection in pre-arrival CXR screening programmes for migrants by TB incidence in country of origin from Aldrige et al. [25].

^b^ NNS = 1/mean prevalence of active TB found through CXR screening stratified by TB incidence in the country of origin.

#### Effectiveness of active tuberculosis treatment

In an ECDC report on TB surveillance from 2007 to 2013, TB treatment outcomes were similar or better in those born outside the EU/EEA than in those born in the EU/EEA [6]. Treatment success was as high in the foreign-born (for all regions of origin) compared with those born in the EU/EEA (77.4% vs 74.6%); however, their failure rates (0.2% vs 2.4%) and default rates (5.4% to 6.6%) were lower. This European surveillance data was judged to be high-quality evidence (Table 1).

#### Acceptability of screening

Mitchell et al. conducted a review to determine the acceptability of targeted TB screening and active case finding among vulnerable and at-risk groups and found that TB screening was well accepted by the majority of risk groups, including migrants (85%; range: 55–96%). Lower acceptability was found among persons living with HIV/AIDS and individuals in refugee camps and internally displaced persons [32]. Overall, the study found that simple TB screening (at point of care) was more acceptable than referral requiring multiple visits. The evidence in this study was judged to have considerable bias (Table 1).

### Cost-effectiveness of active tuberculosis screening programmes

There was very little information on the cost-effectiveness of active TB screening in migrant populations as only three studies were identified. These studies demonstrated that the most cost-effective CXR screening strategies were among high-prevalence groups, close contacts of those with known TB, and migrants at entry if they originated from intermediate (60/100,000) and high (> 120/100,000) TB incidence countries [33-35] (Table 3). 

**Table 3:** Characteristics of included studies for resource use, costs, and cost-effectiveness of active tuberculosis screening

Study	Certainty of economic evidence based on the Drummond criteria ^a^ [27]	Methodological approach/population	Intervention(s)	Cost-effectiveness (ICER or INB) per case prevented	Resource Requirements
Schwartzman et al. 2000 [33]	Certainty of evidence: moderate. Allowance was made for uncertainty in the estimates of costs and consequences, and ranges were provided. No PSA were performed. Justification was provided for a range of values estimated in one-way sensitivity analyses. The cost-effectiveness results were sensitive to model inputs including the probability of INH prescribed; probability of INH treatment completed; cost of inpatient treatment; TB infection rate and HIV seropositivity.	Methods: decision-analytic Markov model; 20 year time horizon; 3% discount rate, perspective of the third-party payer (central and provincial governments); scenario analysis based on INH completion conducted. Population: 20-year-old immigrants to Canada originating from Sub-Saharan Africa, South-east Asia, Western Europe. Cohort 1: 50% TB-positive, 10% HIV-positive. Cohort 2: 50% TB-positive, 1% HIV-positive. Cohort 3: 5% TB-positive, 1% HIV-positive.	Three strategies: (i) No screening (ii) CXR (iii) TST	Cohort 1: TST vs CXR: CAD 2,601(EUR 29,990); CXR vs no screening: CAD 3,934 (EUR 3,618). Cohort 2: TST vs CXR: CAD 66,759 (EUR 61,413); CXR vs no screening: CAD 10,627 (EUR 9,776). Cohort 3: TST vs CXR: CAD 68,799 (EUR 63,289); CXR vs no screening: CAD 236,496 (EUR 217,557).	Resource requirements are high in cohorts 1 and 2, and moderate in cohort 3. Costs/1,000 patients: Cohort 1 (high risk): TST: CAD 436,390 (EUR 401,444); CXR: CAD 338,310 (EUR 311,218); No screening: CAD 332,020 (EUR 305,432). Cohort 2 (intermediate risk): TST: CAD 342,730 (EUR 315,284); CXR: CAD 231,430 (EUR 212,897); No screening: CAD 218,250 (EUR 200,773). Cohort 3 (low risk): TST: CAD 62,640 (EUR 57,623); CXR: CAD 51,170 (EUR 47,072); No screening: CAD 21,820 (EUR 20,072).
Dasgupta et al. 2000 [34]	Certainty of evidence: low. Limited allowance was made for uncertainty in the estimates of costs and consequences; ranges were provided. No PSA was performed No one-way or two-way sensitivity analyses using higher or lower costs, other discount rates or comparisons were performed. Scenario analyses undertaken. The cost-effectiveness results were sensitive to costs for passive diagnosis of TB, INH prescription rate, screening referral criteria and future risk of active TB.	Methods: cost-effectiveness analysis based on prospective non-randomised cohorts; results reported in Canadian dollars; prospective cohort study over 1 year of costs. Population: immigration applicants undergoing CXR screening, already arrived immigrants requiring screening for latent TB, close contacts of active cases resident in Montreal, Quebec, Canada.	Three strategies: (i) CXR in migrants applying for a permanent residence (ii) Surveillance CXR +/− TST (iii) Close contacts CXR +/− TST	Over 1 year, the three programmes detected 27 cases of active TB and prevented 14 future cases. Close-contact screening resulted in net savings of CAD 815 (EUR 758) for each active case detected and treated and of CAD 2,186 (EUR 2,033) for each future active case prevented, compared with passive case detection.	Resource requirements were moderate in applicants and close contacts and higher on those on surveillance. Costs of TB detected and treated: Close contacts CXR +/− TST: CAD 10,275 (EUR 9,560); Applicants CXR: CAD 31,418 (EUR 29,232); Those on surveillance CXR +/− TST: 55,728 (EUR 51,850).
Oxlade et al. 2007 [35]	Certainty of evidence: moderate. Allowance was made for uncertainty in the estimates of costs and consequences; ranges were provided. No PSA was performed One-way or two-way sensitivity analyses using higher or lower costs, other discount rates and test performance characteristics were undertaken. The cost-effectiveness results were sensitive to TST and QFT sensitivity, costs of TST and QFT, close contacts investigation, the passive TB case detection rate and risk of re-activation.	Methods: decision-analytic Markov model; 20 year time horizon; 3% discount rate; Canadian health system perspective; Costs reported in 2004 Canadian dollars. Population: foreign-born entrants to Canada; close contacts of active TB cases.	Five strategies: (i) CXR (ii) No screening (iii) TST (iv) QFT (v) TST followed by QFT if TST-positive	ICER (CAD/case prevented): CXR vs no screening: CAD 875 (EUR 690); TST vs CXR: CAD 9,800 (EUR 7,738), assuming that prescription and completion rates in indicated patients were 100% (relative to the baseline assumption of 73% prescription and 50% completion).	Resource requirement were: low to moderate for CXR and moderate for QFT in immigrants from medium and high incidence countries; high for CXR and QFT in immigrants from low-incidence countries. Costs of CXR screening ranged from: low TB incidence source (2/100,000), CAD 52,553 (EUR 41,499); high TB incidence (120/100,000), CAD 328,190 (EUR 259,160).

CAD : Canadian dollar; CXR: chest radiography; EUR: Euro; HIV: human immunodeficiency virus; ICER: incremental cost-effectiveness ratio; INB: incremental net benefit; INH: isoniazid; PSA: probabilistic sensitivity analysis; QFT: quantiferon; TB: tuberculosis; TST: tuberculin skin test; USD: United States dollar.

^a^ The Drummond Criteria [27]: (i) Was a well-defined question posed in answerable form? (ii) Was a comprehensive description of the competing alternatives given (i.e. can you tell who did what to whom, where and how often)? (iii) Was the effectiveness of the programme or services established? (iv) Were all the important and relevant costs and consequences for each alternative identified? (v) Were costs and consequences measured accurately in appropriate physical units (e.g. hours of nursing time, number of physician visits, lost working days, gained life years)? (vi) Were the cost and consequences valued credibly? (vii) Were costs and consequences adjusted for differential timing? (viii) Was an incremental analysis of costs and consequences of alternatives performed? (ix) Was allowance made for uncertainty in the estimates of costs and consequences? (x) Did the presentation and discussion of study results include all issues of concern to users?

All currencies were converted to 2015 Euros using the Cochrane web-based currency conversion tool: https://eppi.ioe.ac.uk/costconversion/default.aspx. Resource use was expressed in cost per person and classified as low (savings or ≤ USD 1,000/person (EUR 808)), moderate (USD 1,000–100,000/person (EUR 808–80,845)) or high (USD ≥ 100,000/person (EUR > 80,845)).

Two studies demonstrated that CXR screening of migrants was cost-effective compared with no screening: Oxlade et al. determined that the ICER of CXR relative to no screening was CAD 30,000 (Canadian dollars in 2004; EUR 23,690) per case averted in migrants from intermediate TB incidence source countries, and less than CAD 1,000 (EUR 789) per case averted in the high-incidence group [35]. Similarly, CXR compared with no screening in immigrants with a risk of reactivation of more than 5% was cost-effective. Dasgupta et al. reported that close-contact screening resulted in net savings of CAD 815 (EUR 758) for each active case detected and treated and of CAD 2,186 (EUR 2,033) for each future active case prevented, compared with passive case detection [34]. The certainty of the evidence in these studies ranged from low to moderate (Table 1).

## Discussion

There were no single studies that directly addressed the overall effectiveness of active TB screening programmes on the health outcomes of migrant populations. We therefore evaluated the screening chain of evidence. The yield of detecting active TB through CXR screening of migrants was heterogeneous across studies and varied by migrant type and the setting in which the screening was done, but consistently increased with higher TB incidence in the country of origin [25,28,29]. The NNS to detect one case of active TB decreased and cost-effectiveness increased with increasing TB incidence in source countries [25,34,35]. CXR is a highly sensitive and moderately specific screening tool to detect active TB [30,31]. CXR screening is highly acceptable to most foreign-born populations [32].

The yield of CXR to detect active TB varied widely among migrant sub-groups in the three systematic reviews (120 to 2,340/100,000) however the overall yield (350 cases/100,000) in the post-arrival setting was consistent between studies [28,29]. There was also consistency in the increase in yield with increasing TB incidence in source countries in both pre- and post-arrival setting [25,28,29]. The majority of studies in the post-arrival setting were carried out in various EU/EEA countries whereas pre-arrival screening was done in migrants arriving in the United Kingdom. The wide range in yield of post-arrival screening programmes reflects the heterogeneity of the programmes and the composition of migrants screened. Post-arrival programmes differed widely between countries with respect to timing of screening (port of arrival, in reception areas, in the community or ad hoc), countries of origin of migrants received, the type of migrants targeted (all migrants, asylum seekers only or undocumented migrants), and the threshold of TB incidence in the countries of origin at which screening was performed. Although 31 EU/EEA countries have an active TB screening programme for migrants, the absolute and attributable impact on active TB rates in those countries is unknown [37,38]. Extrapolating from the impact of the well-established pre-migration TB programme in the US, there may be benefit of active TB screening in migrants on TB control in the host country. An evaluation of this programme demonstrated that detecting prevalent active TB before arrival in the US reduced TB notification rates among migrants in the first years after arrival [39]. 

Higher NNS and lower cost-effectiveness with higher TB incidence in countries of origin suggests that active TB screening programmes will be most efficient when targeting migrant populations from high TB incidence countries. This is consistent with WHO recommendations to focus active screening on the highest risk groups [40]. The heterogeneity of the estimates from these studies, however, limits the ability to provide more precise guidance on which type of migrants to target, the best timing to screen or the optimal threshold of TB incidence in countries of origin. Although screening migrants from the highest TB incidence countries is most efficient, the impact on TB incidence in the host country might be limited since many cases occur in migrants from countries with lower TB incidence and in migrants who entered the country many years before TB diagnosis [41,42].

Although the CXR is a good screening test for active TB and is highly sensitive (78%), confirmatory sputum culture for TB is essential to improve specificity and is the gold standard for diagnosing active TB [30,31,43]. Screening for symptoms of active TB may be a reasonable first screening tool in certain situations such as in an emergency setting with no on-site CXR facilities. These situations include the reception centres in Italy and Greece and/or when the receipt of a large number of migrants overwhelm health systems (as occurred in Europe in 2015) [8]. Those with symptoms would need referral for CXR. The choice of the screening algorithm will need to be determined by the availability, feasibility and cost of the tests.

Active TB case finding in at-risk populations is an important TB control strategy as it allows for early detection and treatment, reduces individual morbidity and prevents TB spread to others. Active screening programmes are, however, limited by the fact that the yield is low (0.31–1.21%) and that they do not capture or prevent the majority of incident TB cases occurring in the EU/EEA that are primarily due to reactivation of latent TB or new acquisition during travel [13]. Furthermore, the epidemiology of TB in the EU/EEA is heterogeneous. While migrants make up the majority of TB cases in low TB incidence EU/EEA countries, they make up a minority of cases in member states with higher TB incidence (Supplement 2). Screening for active TB in migrants will therefore need to be tailored to the local TB epidemiology in host countries, and the healthcare capacity in each setting [2,3]. Finally, many migrant sub-groups are vulnerable and face barriers in accessing heath care and treatment in the EU/EEA [44]. Addressing barriers in accessing care and treatment for all migrants, including the right to healthcare access for all and programmes tailored to address unique needs, will be essential to ensuring the most effective active TB screening and treatment programmes. 

### Study limitations

Our study was limited by the fact that we did not retrieve any studies that directly estimated the effectiveness of active TB screening and by the very limited data on the cost-effectiveness of active TB screening. The search was limited by the fact that it was conducted up until May 2016 and that we only included studies published in English or French. A recent narrative review of the effectiveness and cost-effectiveness, however, reports similar literature and findings as our study [45]. Our findings are further limited by the quality of the original studies that were included in the systematic reviews. Study quality was low or very low, as almost all included studies were observational studies.

### Evidence gaps and future directions

Robust studies on the yield of active TB screening among migrants by age group, migration type, timing of screening, threshold of TB incidence in source countries and the associated cost-effectiveness will be required to design the most effective active TB screening programmes. Additional studies are needed that determine the absolute and attributable impact of active TB programmes on TB control in low-incidence countries in the EU/EEA and the optimal threshold of incidence in source countries at which to screen. Finally, evidence on the comparative effectiveness and cost-effectiveness of different TB control strategies (active vs latent TB screening) for migrants will be required to prioritise TB control efforts for this population.

## Conclusions

Active TB screening programmes that target migrants from high TB incidence countries will provide the highest yield and will be the most cost-effective. The heterogeneity of the estimates from the studies identified and the small number of studies addressing both the effectiveness and cost-effective of active TB screening in migrants limits the ability to provide precise guidance on which type of migrants to target, the best timing to screen or the optimal threshold of TB incidence in countries of origin. This highlights the need for further data to inform active TB screening programmes for migrants in the EU/EEA.
